# SS-OPDet: A Semi-Supervised Open-Set Detection Framework for Dead Pine Wood Detection

**DOI:** 10.3390/s25113407

**Published:** 2025-05-28

**Authors:** Xiaojian Lu, Shiguo Huang, Songqing Wu, Feiping Zhang, Mingqing Weng, Jianlong Luo, Xiaolin Li

**Affiliations:** 1 College of Computer and Information Sciences, Fujian Agriculture and Forestry University, Fuzhou 350002, China; 17759636657@163.com (X.L.); sghuang@fafu.edu.cn (S.H.); 5221139013@fafu.edu.cn (J.L.); 2 College of Forestry, Fujian Agriculture and Forestry University, Fuzhou 350002, China; dabinyang@126.com (S.W.); fpzhang1@163.com (F.Z.); 15568821186@163.com (M.W.)

**Keywords:** semi-supervised learning, open-set detection, dead pine wood, weighted multi-scale feature fusion, dynamic confidence pseudo-label generation

## Abstract

Pine wilt disease poses a significant threat to pine forests worldwide, necessitating efficient and accurate detection of dead pine wood for effective disease control and forest management. Traditional deep learning methods based on supervised closed-set paradigms often struggle to address unknown interfering objects, causing false positives, missed detection, and increased annotation burdens. To overcome these challenges, we propose SS-OPDet, a semi-supervised open-set detection framework that leverages a small amount of labeled data along with abundant unlabeled data. SS-OPDet integrates a Weighted Multi-scale Feature Fusion module to dynamically integrate global- and local-scale features, thereby significantly improving representational accuracy for dead pine wood. Additionally, a Dynamic Confidence Pseudo-Label Generation strategy categorizes predictions by confidence level, effectively reducing training noise and maximizing the use of reliable unlabeled data. Experimental results from 7733 UAV images demonstrate that SS-OPDet achieves an average precision (${AP}_{K}$) of 84.73%, a recall ($R_{K}$) of 94.48%, an Absolute Open-Set Error (*AOSE*) of 271 and a Wilderness Impact (*WI*) of 0.0917%. Cross-region validation further confirms the robustness and generalization capability of the proposed framework. The proposed method offers a cost-effective and accurate solution for timely detection of pine wilt disease, providing substantial benefits to forest monitoring and management.

## 1. Introduction

Pine wilt disease (PWD), known for its rapid spread and high mortality rate, poses a significant threat to global pine forest ecosystems and has become one of the major forest diseases [1]. Timely detection and removal of infected trees are essential to prevent rapid disease proliferation, which can lead to extensive ecological degradation and significant economic losses [2]. In recent years, the widespread adoption of unmanned aerial vehicle (UAV) remote sensing and advancements in image resolution have enabled researchers to rapidly acquire high-resolution imagery across extensive forested areas, thereby facilitating the efficient and accurate detection of dead pine wood [3,4].

In this context, deep learning has emerged as a pivotal tool for automatically extracting and handling large-scale image datasets. Its applications in dead pine wood detection have been extensively explored. Numerous studies have introduced methodological enhancements to address the inherent challenges associated with remote sensing-based detection of dead pine wood. For instance, to enable UAV-based dead pine wood detection, Bai et al. [5] enhanced the Mamba model by integrating an attention mechanism. Their method integrates an SSM-centric Mamba backbone with a Path Aggregation Feature Pyramid Network for multi-scale feature fusion and employs depth-wise separable convolutions to enhance convolution efficiency. Similarly, Wang et al. [6] developed a large-scale dead pine wood detection dataset by combining Digital Orthophoto Map and Digital Surface Model information. By leveraging spectral and terrain features of remote sensing data, their method effectively reduces the misclassification of ambiguous targets. In a related study, Li et al. [7] employed an enhanced Mask R-CNN integrated with a prototype network to support the intelligent identification of individual trees infected by PWD. Furthermore, Cai et al. [8] fused high-resolution UAV RGB imagery with Sentinel-2 multispectral data to develop a YOLOv5-PWD-based model for detecting individual trees in PWD-affected forests, leveraging data augmentation techniques to improve detection performance. In addition, Wang et al. [9] proposed a semi-supervised semantic segmentation model, GAN_HRNet_Semi, based on generative adversarial networks, which integrates expansion prediction, grid vectorization, and forest bottom mask extraction strategies, providing a novel solution for precise and large-scale monitoring of PWD. Han et al. [10] produced confidence maps using Gaussian kernels, incorporated a multi-scale spatial attention mechanism, and applied a copy–paste augmentation technique to accurately detect dead pine wood from aerial remote sensing imagery affected by PWD. Lastly, You et al. [11] developed a general deep learning-based general system utilizing RGB UAV orthophotos for dead pine wood detection, thereby enabling precise and efficient disease monitoring.

**Figure 1:**
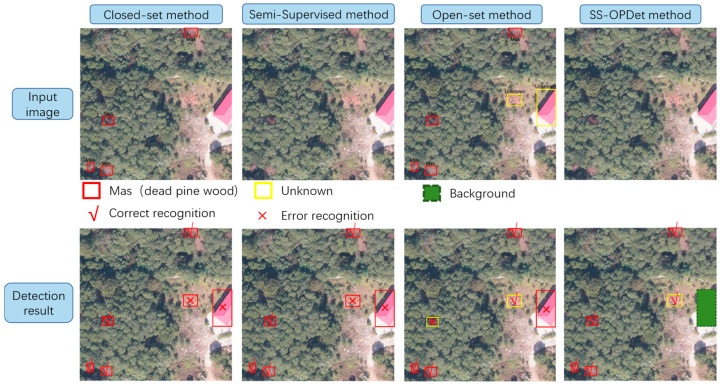
Comparison of different methods for dead pine wood detection.

Although modern deep learning methods have significantly improved the accuracy and efficiency of dead pine wood detection, most methods are still based on a closed-set object detection paradigm that assumes identical target categories in both the training and testing sets. As shown in the first column of Figure 1, the detection results include only the dead pine wood class from the input image. However, in real UAV imagery, unannotated interfering objects—such as loess, buildings, and fallen trees—often appear alongside dead pine wood. This discrepancy limits the model’s ability to learn the characteristics of unknown categories during training. As a result, the model may misclassify unknown objects as dead pine wood or erroneously label actual dead pine wood as background, leading to increased false positives and false negatives that significantly degrade detection performance. For example, the red crosses in the red box in the first column of the second row in Figure 1 highlight cases where unknown categories, such as loess and buildings, are incorrectly identified as dead pine wood. In addition, in UAV imagery, the demand for large-scale annotated datasets has become increasingly urgent, placing substantial burden on manual annotation efforts.

To reduce dependence on large volumes of annotated samples, researchers have proposed semi-supervised object detection (SSOD) methods. These methods leverage a limited set of labeled data together with a large pool of unlabeled data (e.g., images without annotations, as shown in Figure 1, first row, columns 2 and 4), thereby enabling effective learning across diverse data distributions through strategies such as pseudo-label generation, self-consistency regularization, and teacher–student frameworks. This paradigm significantly alleviates the manual annotation. For example, Huang et al. [12] adopted a two-stage hierarchical semi-supervised learning framework to optimize the YOLOv7 model via a semi-supervised sample mining strategy, successfully achieving the extraction of individual trees infected by pine wood nematode. Ben et al. [13] first generated preliminary tree crown training data using LIDAR-based unsupervised detection, and then fine-tuned a RetinaNet-based RGB tree crown detector using a small set of high-quality manual annotations, enabling efficient extraction of individual crowns in forest environments. Wang et al. [9] utilized a GAN-based semi-supervised deep semantic segmentation framework that incorporates expansion prediction, grid vectorization, and forest mask extraction techniques to accurately identify standing trees exhibiting color changes caused by pine wilt disease. In a separate study, Zhao et al. [14] employed a cascade detector with Focal Loss and SmoothL1 Loss, achieving a 1.6% AP improvement over the Combating Noise method. Luo et al. [15] proposed a semi-supervised framework that integrates a channel-prior convolutional attention mechanism and an adaptive thresholding strategy based on a Gaussian mixture model, and applied it to UAV imagery for detecting dead pine trees in the context of pine wilt disease prevention and control, providing a low-cost and efficient monitoring solution. Finally, Vennervirta et al. [16] employed a semi-supervised object detection network to identify collapsed dead pine wood. Although the detection rate was relatively low, their findings emphasized the significant influence of site conditions, vegetation characteristics, and image resolution on detectability, thereby providing valuable insights for future improvements in dead wood detection.

However, most existing semi-supervised methods still rely on the closed-set assumption, making them prone to misclassifying unknown categories as dead pine wood. For example, as shown by the red cross in the red box in the second row, in the second column of Figure 1, unknown objects such as loess and buildings are incorrectly identified as dead pine wood. To overcome the limitations of this assumption when dealing with unknown targets, open-set object detection has been introduced [17,18,19,20,21,22,23]. This approach trains exclusively on known categories. As illustrated in the first row, third column of Figure 1, the input image includes both dead pine wood and unknown objects; however, only the known class (dead pine wood) is used for supervision during training. During inference, when the model encounters unseen objects, it classifies them as “unknown” or background. As shown by the red check mark in the red box in the second row, third column of Figure 1, loess is correctly detected as an unknown category. Although this mechanism provides clear benefits in handling visually similar but novel targets, detection errors may still occur—for instance, as indicated by the yellow box with a yellow cross in the second row, third column, where dead pine wood is misclassified as unknown, and in the red box with a red cross, unknown categories are incorrectly detected as dead pine wood. Moreover, current open-set detection methods still rely on large volumes of annotated data to maintain detection performance for known categories, posing a considerable challenge in the era of large-scale image annotation. Consequently, semi-supervised open-set object detection is gradually becoming a new research focus [24,25,26,27]. Its primary objective is to fully leverage unlabeled data to enhance the model’s learning capacity, while introducing effective mechanisms to distinguish unknown categories or treat them as background, thereby reducing false positives and missed detections. As shown in the fourth column of the second row in Figure 1, all dead pine wood targets are correctly detected, and the previously misidentified unknown objects are correctly treated as background (green box). Hence, applying a semi-supervised open-set object detection method to dead pine wood detection is considered essential for improving detection robustness and reducing annotation costs.

To demonstrate the innovation of our proposed SS-OPDet framework and simultaneously evaluate its applicability and robustness in real forest environments, we constructed a large-scale dataset capturing the spatial distribution of dead pine wood. The dataset contains 29,575 samples of dead pine wood and 7334 samples of unknown categories, collected from multiple regions under diverse environmental conditions. Additionally, detailed cross-region experiments were conducted to mitigate spatial autocorrelation and validate the generalization capability of the model. Our main contributions are as follows:

(1) First Application of Semi-Supervised Open-Set Detection for Dead Pine Wood Recognition: Conventional methods based on closed-set assumptions or semi-supervised strategies are inadequate for effectively handling unknown interfering targets. By reducing the quantity of annotated data by half and introducing an “unknown” (or background) classification strategy, our approach effectively excludes novel, unseen categories, thereby significantly reducing both false positives and false negatives.
(2) Introduction of Two Novel Modules—Weighted Multi-scale Feature Fusion (WMFF) Module and Dynamic Confidence Pseudo-Label Generation (DCPL) Strategy: The WMFF module performs weighted fusion of multi-scale feature maps to highlight the most discriminative features for dead pine wood detection. At the same time, the DCPL strategy divides predicted bounding boxes into high, medium, and low confidence levels, thereby enhancing pseudo-label quality and effectively reducing label noise.
(3) Cross-Regional Validation and Adaptation Testing: Cross-regional experiments were conducted to evaluate the adaptability and generalization capability of SS-OPDet under varying forest conditions. By sequentially designating five distinct regions as test sets, our experimental results show that the proposed methods consistently achieve excellent detection performance across diverse environments, providing robust technical support for early intervention in pine wilt disease and related detection applications.

## 2. Materials and Methods

### 2.1. Dataset Acquisition

**Figure 2:**
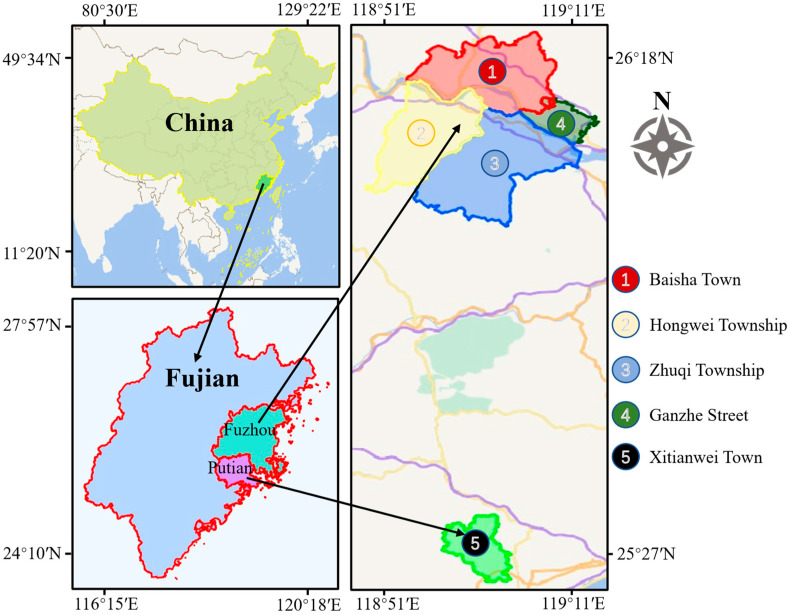
Dead pine wood data collection areas in Minhou County, Fuzhou City and Putian City, Fujian Province.

This study aims to detect dead wood in pine forests to support prevention, treatment, and forest management efforts. To this end, we collected dead pine wood imagery over different periods to evaluate the feasibility of SS-OPDet. We manually constructed the dataset used in this study, covering multiple townships in Minhou County, Fuzhou City, and Putian City, Fujian Province. The coordinate range extends from 25°27′ to 26°18′ N and from 118°51′ to 119°11′ E, covering a total area of 15,400 hectares. Specifically, the data collection areas include Baisha Town (approximately 7467 ha), Hongwei Township (5333 ha), Zhuqi Township (1133 ha), Ganzhe Street (933 ha) in Minhou County, and Xitianwei Town (533 ha) in Xianyou County, Putian City. Figure 2 shows the data collection areas. For image acquisition, we utilized a CW-007 fixed-wing drone (Chengdu JOUAV Automation Tech Co., Ltd., Sichuan, China) equipped with a CA-102 42-megapixel camera, with data collected from November 2020 to October 2021. Due to terrain constraints, the flight altitude was maintained between 800 and 1200 m, and the image resolution was set at 7952 × 5304 pixels. All images were stitched into 39 TIF images using Pix4DMapper software (version 4.6.3), with sizes ranging from 31,984 × 26,045 pixels to 64,033 × 50,719 pixels. Subsequently, these images were cropped into 600 × 600-pixel sub-images, resulting in a total of 8598 sub-images.

### 2.2. Data Preprocessing

**Figure 3:**
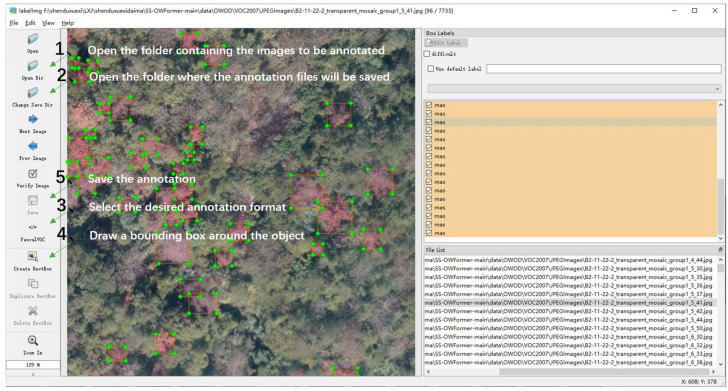
Example of manual annotation for both dead pine wood and unknown category objects using the LabelImg tool.

**Figure 4:**
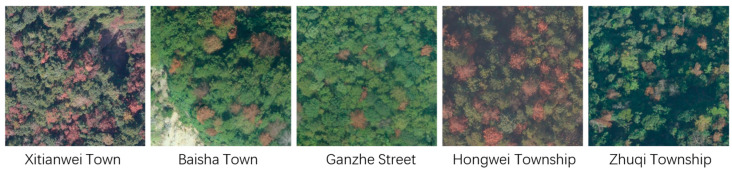
Sample images from the constructed dataset.

Dataset diversity is widely recognized as a key factor influencing model performance. Image entropy, which quantifies the level of texture information or randomness in an image, is commonly used to assess image quality by identifying low-information regions. Following the approach of Wang et al. [28], we employed image entropy to filter out blank and edge images with entropy values below 5. To ensure real-world variability and enhance model robustness, approximately 71% of the images were collected under clear skies, 7% under overcast conditions, and 22% during dusk or other low-light scenarios. Additionally, several overexposed and backlit images were intentionally retained to further increase dataset diversity. After screening, a total of 7733 images were selected and annotated. Among the 7733 selected images, approximately 6.1% of the images were collected from Xitianwei Town, 45.6% from Baisha Town, 27.9% from Hongwei Township, 11.5% from Zhuqi Township, and 8.9% from Ganzhe Street, which included 29,575 instances of dead pine wood. Additionally, unknown category objects in the images were annotated, resulting in 7334 instances of unknown categories. Both dead pine wood and unknown objects were manually annotated using the LabelImg tool, as shown in Figure 3. For each object, the minimum bounding rectangle was marked as the detection box, and the annotations were saved in XML format. The XML files recorded each object’s category, height, width, and the coordinates of the top-left and bottom-right corners of the bounding box. Initially annotated in VOC format, the dataset was later converted into COCO format and partitioned into training, validation, and test sets. In the earlier experiments, we randomly split the full dataset into training/validation and testing sets in a 9:1 ratio. Then, the training/validation set was further divided into training and validation subsets in a 6:4 ratio. To further evaluate the applicability and generalization capability of the constructed dataset for the SS-OPDet under varying forest conditions, we adopted a cross-region testing scheme in the subsequent experiments. Specifically, the entire dataset was divided by geographical location into five non-overlapping regions, each encompassing multiple imaging conditions to ensure both diversity and representativeness (see Figure 4). In the experiments, we employed a leave-one-region-out cross-validation strategy, where each round one region was selected as the test set and the remaining regions were used for training and validation. Each test region was designated as an individual test set, while the remaining regions were merged and split into training and validation subsets using the same 6:4 ratio. This approach aimed to assess the model’s detection performance on entirely unseen regional data and its robustness to environmental variability across regions. Evaluation metrics included common measures such as ${AP}_{K}$ and $R_{K}$. This strategy is expected to demonstrate that SS-OPDet maintains stable and high detection accuracy across diverse forest conditions, thereby confirming its broad applicability for early intervention in pine wilt disease and related forest management tasks.

### 2.3. Methods

**Figure 5:**
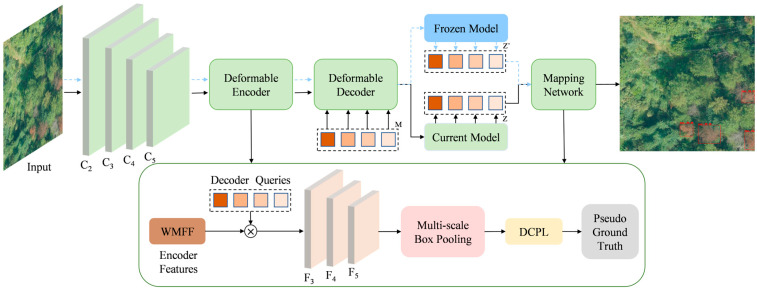
SS-OPDet network architecture.

To reduce the misclassification of unknown targets as dead pine wood and to minimize both false negatives and false positives, we propose a Semi-Supervised Open-Set Detection of Dead Pine Wood (SS-OPDet) framework. Figure 5 illustrates the overall architecture of the proposed SS-OPDet framework. The blue dashed arrow represents the flow from the Frozen Model, and the black solid arrow represents the flow from the Current Model. The input image is first passed through the backbone to extract multi-scale features, which are then processed by the Deformable Encoder to capture target information at multiple spatial scales via deformable attention mechanisms. In the Deformable Decoder, the Decoder Queries interact with the encoder outputs to preliminarily generate bounding box and class predictions for targets. In the semi-supervised incremental learning process, the model concurrently maintains a Current Model and a Frozen Model: the target queries Z’ produced by the Frozen Model are aligned to the query vectors Z of the Current Model through a Mapping Network, thereby smoothly transferring existing knowledge from the old task to the new one. During the feature extraction stage, a WMFF module is also incorporated to adaptively emphasize the most discriminative channels and scales for dead pine wood detection during multi-scale feature integration. The multi-scale prediction results are uniformly aggregated via Multi-scale Box Pooling, which selects the optimal positions and classes from detection boxes across different scales. For unlabeled images, the detection boxes produced by the decoder are categorized into high, medium, and low tiers based on their confidence scores by the DCPL strategy, with only high- and medium-confidence boxes regarded as pseudo ground truth for training, thus reducing the adverse impact of noisy interference. Overall, by leveraging a small amount of labeled data alongside a large set of unlabeled images, the network achieves precise learning of the known “dead pine wood” class while effectively excluding unknown interference objects, thereby realizing semi-supervised dead pine wood detection in an open-set environment.

#### 2.3.1. Weighted Multi-Scale Feature Fusion

In semi-supervised open-set dead pine wood detection, the model must accurately distinguish subtle differences between dead pine wood and interfering targets (such as loess, buildings, and fallen trees) while effectively leveraging both local details and global semantic information across different scales. Traditional multi-scale processing methods typically employ fixed resolutions or directly concatenate feature maps. This approach fails to effectively balance the heterogeneity and complementarity of information at various scales—especially in complex forest environments where targets have similar appearances—which can lead to missed detection of small targets and false positives for interfering objects [29]. To address this issue, we propose the WMFF module, which dynamically fuses multi-scale feature maps extracted by a Transformer encoder, thereby integrating cross-scale information and significantly enhancing both the detection accuracy and robustness in dead pine wood detection.

**Figure 6:**
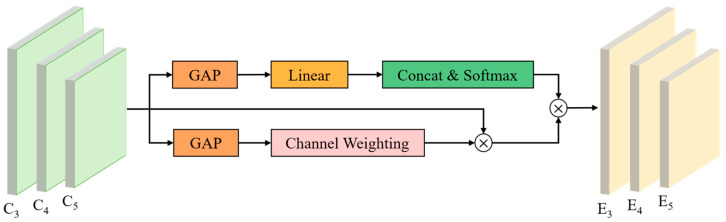
WMFF module structure.

The structure of the WMFF module is shown in Figure 6. It consists of two parallel branches designed to compute attention weights from different perspectives. The upper branch applies global average pooling (GAP), followed by a linear transformation and softmax activation, to generate scale-level importance scores, allowing the model to assess which feature scales contain the most informative semantics. The lower branch performs channel-wise GAP and incorporates a channel attention mechanism to evaluate the local discriminability of features within each scale. The two attention maps are subsequently used to re-weight the original feature maps, which are then fused to produce an enhanced multi-scale representation. This design enables the model to simultaneously focus on both scale-specific and channel-specific patterns, which are essential for distinguishing dead pine wood from visually similar background elements.

Let the i-th scale feature map from the Transformer encoder be denoted as

(1) $$F_{i}\in R^{H_{i}\times W_{i}\times C},$$

where $H_{i}$ and $W_{i}$ represent the height and width of the feature map, and *C* is the number of channels.

To achieve adaptive weighting during fusion, we first perform global average pooling (GAP) on each scale to obtain its global descriptor vector

(2) $$v_{i}=\frac{1}{H_{i}W_{i}}\sum_{h=1}^{H_{i}}\;\sum_{w=1}^{W_{i}}\;F_{i}(h,w),$$

where $v_{i}$ is the global statistic for scale *i* and $F_{i}(h,w)$ is the channel vector at position (h,w) in $F_{i}$.

Next, a dedicated linear mapping (i.e., a fully connected layer) is applied to $v_{i}$ to produce a scalar $s_{i}$

(3) $$s_{i}={FC}_{i}(v_{i}),$$

where $s_{i}$ denotes the overall activation intensity of scale i in the current image, and FC() maps the dimension $v_{i}$ to 1.

All scalars $s_{i}$ (for each scale) are then concatenated and normalized using a *softmax* function to obtain the dynamic ratio $\lambda_{i}$.

(4) $$[\lambda_{3},\lambda_{4},\lambda_{5}]=softmax([s_{3},s_{4},s_{5}]),$$

where $\lambda_{i}$ indicates the overall importance of scale i in the final fusion.

To further emphasize the local channel-level differences of scale i, we compute a local weighting coefficient $w_{i}$ for the feature map F. This is given by

(5) $$w_{i}=\sigma \left(\frac{1}{H_{i}W_{i}}\sum_{h=1}^{H_{i}}\;\sum_{w=1}^{W_{i}}\;F_{i}(h,w)\right),$$

where $w_{i}$ is used to measure the overall activation level of the channels in scale i for the current image.

Subsequently, the feature map $F_{i}$ is modulated by the local weighting coefficient:

(6) $${\tilde{F}}_{i}=w_{i}\cdot F_{i},$$

Finally, each scale’s modulated feature map is weighted by its corresponding dynamic ratio to obtain the final weighted output

(7) $${\hat{F}}_{i}=\lambda_{i}\cdot {\tilde{F}}_{i},$$

By combining local weighting and global dynamic scaling, the WMFF module emphasizes critical differences between dead pine wood and background interference across multiple scales, thereby significantly improving the model’s discriminative ability for small targets and complex scenes.

In dead pine wood detection tasks, the targets are typically small and exhibit high visual similarity to the background. By calibrating both local and global weights, the WMFF module ensures that the most discriminative scale-level features are more prominently represented after fusion, thereby significantly reducing the risks of false positives and false negatives. This method effectively preserves the detailed features of dead pine wood while suppressing background noise when confronted with highly similar interfering objects such as loess, buildings, and fallen trees. Consequently, it provides more discriminative feature representations for the subsequent decoding stage, thereby further improving overall detection accuracy and robustness.

#### 2.3.2. Dynamic Confidence-Based Pseudo-Labeling

Although the WMFF module enhances the model’s ability to capture fine details of dead pine wood through Weighted Multi-scale Feature Fusion, relying solely on this mechanism does not fully address the challenges associated with pseudo-label generation in semi-supervised open-set dead pine wood detection. Unlabeled images generate a large number of candidate boxes, and adaptively selecting high-quality and representative pseudo-labels is critical for achieving optimal detection performance. Traditional methods typically adopt a fixed threshold or a Top-K strategy, in which all predicted boxes are ranked by confidence scores and a fixed number of the top-scoring boxes are selected as pseudo-labels [30]. However, this approach presents two major limitations. First, a fixed number of candidate boxes cannot adapt to the varying confidence score distributions across different image batches; second, when dead pine wood targets exhibit visual similarity to interfering objects such as loess, buildings, and fallen trees, some potential true targets may be discarded due to not reaching a high confidence threshold, while some interfering targets with relatively high scores may be erroneously included as pseudo-labels, leading to increased false positives and false negatives.

To address these issues, we propose a DCPL strategy, which sets high and low confidence thresholds to adaptively divide all predicted boxes into three intervals—high, medium, and low confidence. During training, only candidate boxes in the high and medium confidence intervals are retained as pseudo-labels. This strategy not only preserves sufficient potential positive samples but also effectively eliminates noisy candidates, thereby enhancing the overall quality of the model training process.

**Figure 7:**
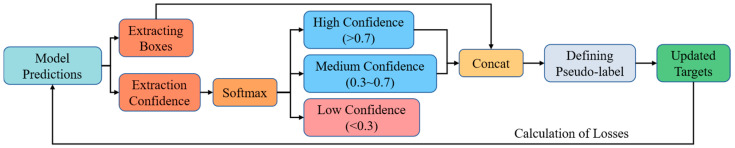
DCPL strategy structure.

Figure 7 shows the overall process of the proposed DCPL strategy. Unlike previous methods that apply a single confidence threshold or a Top-K rule, DCPL categorizes model predictions into three confidence levels—high, medium, and low—based on adaptive thresholding. Boxes with high and medium confidence are retained as pseudo-labels, while low-confidence predictions are discarded. This three-tier confidence mechanism mitigates the risk of over-reliance on high-confidence false positives and the exclusion of valuable low-confidence true positives. It introduces a soft transition zone between clear positives and negatives, thereby improving pseudo-label quality and training stability under open-set uncertainty.

Assume that the raw class scores output by the model during inference are normalized via *softmax*, denoted as

(8) $$\hat{p}=softmax(pred\_logits),$$

where $\hat{p}\in R^{M\times (K+1)}$ denotes the probability distribution over *K* + 1 classes for the M-th predicted box, and pred_logits are the raw class scores output by the network.

Let ${\hat{p}}_{max}=\underset{c}{max}\;(\hat{p})$ denote the maximum confidence score across the c-th class. A predicted box is classified as follows:

High-confidence box: ${\hat{p}}_{max}>high\_conf\_thresh$.

Low-confidence box: ${\hat{p}}_{max}>low\_conf\_thresh$ (these boxes are directly discarded).

Medium-confidence: box: $low\_conf\_thresh\leq {\hat{p}}_{max}\leq high\_conf\_thresh$.

Let $\hat{b}$ denote the predicted bounding boxes output by the network. For all boxes that meet the high- and medium-confidence conditions, we merge them as

(9) $$\mathrm{pseudo}\_\mathrm{boxes}={\hat{b}}_{high\_conf}\cup {\hat{b}}_{mid\_conf},$$

We then assign a unified class label of −1 to these boxes. To incorporate these pseudo-labels for the next training update, they are merged into the existing set of target labels, targets, forming a new target label set:

(10) targets’=targets∪(pseudo_boxes,−1),

This dynamic partitioning strategy not only ensures the retention of high-confidence candidate boxes corresponding to dead pine wood but also preserves medium-confidence candidates that may contain valid information, thereby effectively reducing the risk of false negatives. Low-confidence candidate boxes typically correspond to background or interfering objects that clearly lack the characteristics of dead pine wood; discarding these candidates helps to mitigate the adverse effects of accumulating pseudo-label noise during training. As the model’s feature representation capability improves during training, some medium-confidence candidates may receive higher confidence scores in subsequent training iterations and gradually transition into high-confidence candidates, whereas those that consistently fail to meet the criteria will be classified as background or assigned to the unknown category and subsequently discarded.

Compared with the traditional fixed Top-K strategy, the DCPL strategy demonstrates greater flexibility in adapting to variations in confidence score distributions across different image batches. The fixed “Top-K” selection approach often fails to capture moderately confident candidate boxes that may still be informative, whereas a single high-threshold screening method tends to overlook potential targets. By setting both high and low thresholds simultaneously, DCPL not only ensures higher detection accuracy but also maximizes the retention of potentially valid dead pine wood candidate true boxes.

### 2.4. Model Training and Evaluation

#### 2.4.1. Training Environment and Parameters

Dataset: A custom-constructed dead pine wood dataset was used in this study. Specifically, the dataset comprises 7733 fully annotated images of dead pine wood, most of which do not include interfering targets such as buildings, loess, or fallen trees. A small subset of the images includes annotations for all target categories. The dataset was divided into training, validation, and test sets. Training images were curated to contain annotations exclusively for dead pine wood, meaning that training was performed on this single category. Validation and testing, however, were carried out on all categories, including the unknown ones such as buildings, loess, and fallen trees.

Implementation Details: The experiments were conducted on Ubuntu 18.04 using a single NVIDIA GeForce RTX 3090 24G GPU, Compute Unified Device Architecture (CUDA) 11.3, and an Intel^®^ Xeon^®^ CPU E5-2678. All models were trained in the same settings. Specifically, the training of SS-OPDet utilized 50% of the annotated data, with the following parameter settings: an initial learning rate of 0.0002, a learning rate of 0.00002 for the backbone network, a batch size of 2, and a weight decay of 0.0001.

#### 2.4.2. Evaluation Metrics

In our study on semi-supervised open-set dead pine wood detection, $P_{K}$ denotes the precision for the “dead pine wood” category. We also adopt the Absolute Open-Set Error (*AOSE*) [31] to quantify the number of unknown category instances that are mistakenly classified as known; in this context, *AOSE* specifically measures the number of unknown instances misclassified as “dead pine wood”. Furthermore, we report the average precision for the “dead pine wood” category (${AP}_{K}$), which assesses the proportion of correctly predicted “dead pine wood” instances among all predicted instances labeled as such. In addition, we report the recall $\left(R_{K}\right)$ for the “dead pine wood” category, which measures the proportion of “dead pine wood” instances that are correctly detected, and also use the Wilderness Impact (*WI*) [18] as a metric to measure the extent to which unknown targets are misclassified as known categories, providing a fine-grained assessment of open-set robustness. The evaluation formulas are defined as follows:

(11) $$P=\frac{TP}{TP+FP},$$

(12) $$R=\frac{TP}{TP+FN},$$

(13) $$AP=\int_{0}^{1}\;P(R)dR,$$

(14) $$WI=\frac{P_{K}}{P_{K\cup U}}-1$$

where *P* is the precision, representing the ratio of true positive detections (*TP*) to the sum of true positives and false positives (*FP*). *TP* is the number of correctly detected dead pine wood instances. *FP* is the number of instances erroneously identified as dead pine wood. *R* is the recall, representing the ratio of true positive detections to the sum of true positives and false negatives (*FN*). *FN* is the number of dead pine wood instances that the model fails to detect. *AP* is computed as the area under the precision–recall curve, summarizing the model’s precision across all recall levels. *K* denotes the number of known categories (in this study, *K* = 1). *U* denotes the number of unknown categories (in this study, *U* = 1). $P_{K}$ is the precision for the known category, specifically for “dead pine wood”. $P_{K\cup U}$ is the precision considering both known and unknown categories, measuring the proportion of correctly identified instances when both types are included.

## 3. Results

### 3.1. Overall Performance Comparison

In this study, SS-OPDet exhibited superior performance in the task of dead pine wood detection. To verify the effectiveness of SS-OPDet, we compared it with several state-of-the-art methods, including OW-DETR [32] and SS-OWFormer. This experimental setup was designed to comprehensively evaluate the performance advantages of SS-OPDet in dead pine wood detection tasks.

In addition, SS-OPDet was further compared with recent fully supervised open-set detection methods, including OpenDet and Grounding DINO, to evaluate its performance under open-set conditions.

#### 3.1.1. Quantitative Analysis

As shown in Table 1, SS-OPDet outperforms all other semi-supervised open-set detection methods across all evaluation metrics. Specifically, SS-OPDet achieves an ${AP}_{K}$ of 84.73%, which is 2.3 percentage points higher than SS-OWFormer’s 82.44% and 3.39 points higher than OW-DETR’s 81.34%, demonstrating a significant advantage in detecting known dead pine wood targets. Moreover, SS-OPDet attains an $R_{K}$ of 94.48%, which is 1.67 percentage points higher than SS-OWFormer’s 92.81%, confirming its superior ability to retrieve known targets. In terms of open-set robustness, SS-OPDet achieves the lowest *AOSE* of 271, compared to 305 for SS-OWFormer and 433 for OW-DETR. This demonstrates its stronger capability to exclude unknown targets and avoid false activations. Furthermore, SS-OPDet attains the lowest *WI* of 0.0917%, compared to 0.0958% for SS-OWFormer and 0.2131% for OW-DETR. These results confirm that SS-OPDet not only improves detection of known categories but also excels in suppressing misclassification of unknown and confusing background objects (e.g., loess, buildings) as dead pine wood. In summary, SS-OPDet demonstrates a more balanced and robust performance than other semi-supervised open-set detection baselines, especially under complex forest conditions.

Simultaneously, we include two recent and representative open-set object detection methods—OpenDet and Grounding DINO—for comparison. These models operate under full supervision (100% labeled data) and achieve high ${AP}_{K}$ values (86.48% and 87.35%, respectively). However, they exhibit significantly higher *AOSE* (386 and 352) and *WI* (2.6406% and 2.2875%), indicating a reduced ability to reject unknown targets and a higher tendency to misclassify background objects. In contrast, our SS-OPDet, even with only 50% labeled data, achieves lower *AOSE* (271) and the lowest *WI* (0.0917%) among all models tested. This highlights the superior open-set robustness and noise suppression capabilities of SS-OPDet, demonstrating its effectiveness in complex real-world scenarios where both label efficiency and unknown rejection are critical.

#### 3.1.2. Qualitative Analysis

**Figure 8:**
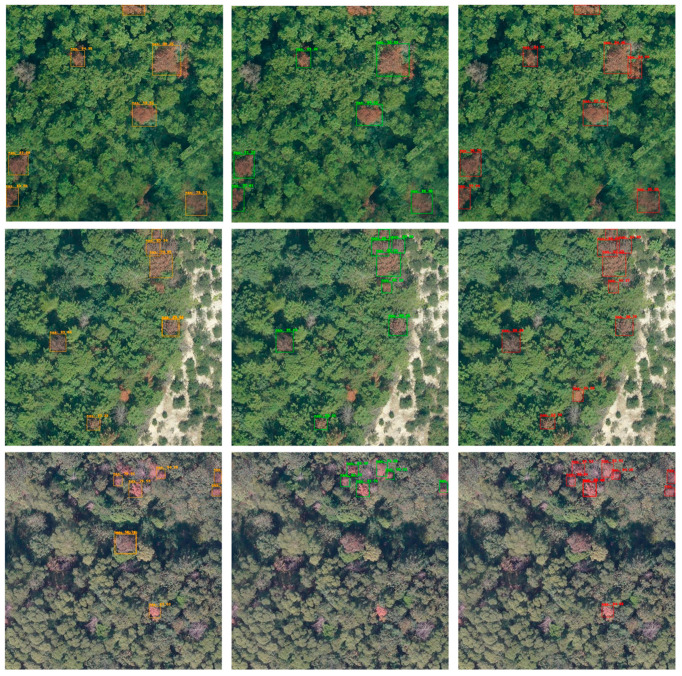

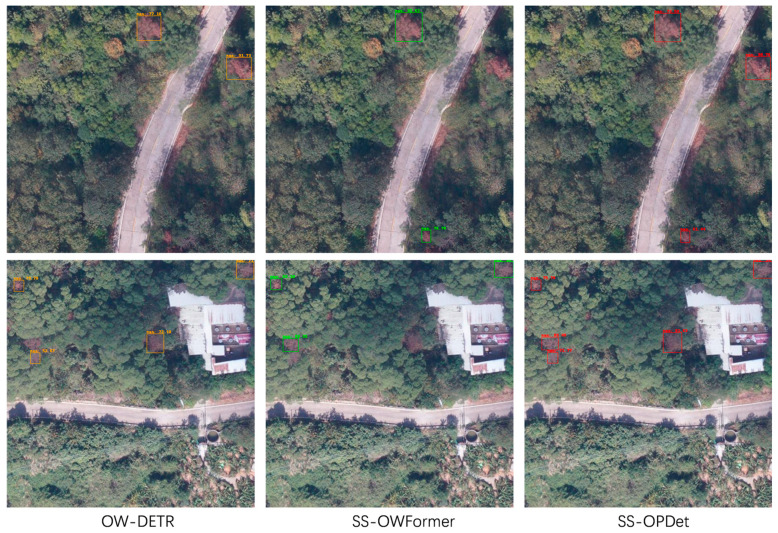
Comparison of dead pine wood detection results for different methods.

Figure 8 illustrates a visual comparison of dead pine wood detection results across five representative scenes for three methods: OW-DETR, SS-OWFormer, and SS-OPDet. In this figure, orange boxes represent the detection results of OW-DETR, green boxes represent those of SS-OWFormer, and bright red boxes represent the results of SS-OPDet. Each row corresponds to a distinct scene, with the left, middle, and right columns displaying the detection outputs of OW-DETR, SS-OWFormer, and SS-OPDet, respectively. It can be seen that the orange bounding boxes in the left column (OW-DETR) and the green boxes in the middle column (SS-OWFormer) successfully identify some dead pine wood targets, yet both methods exhibit noticeable missed detection and false positives in multiple scenes. In contrast, the red bounding boxes in the right column (SS-OPDet) show a more complete and accurate detection across all five scenes, with minimal missed detection. The enhanced performance of SS-OPDet stems from its optimized design for semi-supervised open-set detection, which integrates two key modules: the WMFF module, for adaptive multi-scale feature fusion, and the DCPL strategy, for robust pseudo-label generation. Through the synergistic effect of these two modules, SS-OPDet effectively leverages the limited labeled data and abundant unlabeled data to accurately exclude unknown categories and precisely detect known targets, ultimately achieving superior detection performance across diverse scenes compared to the other methods.

#### 3.1.3. Quantitative Analysis

**Figure 9:**
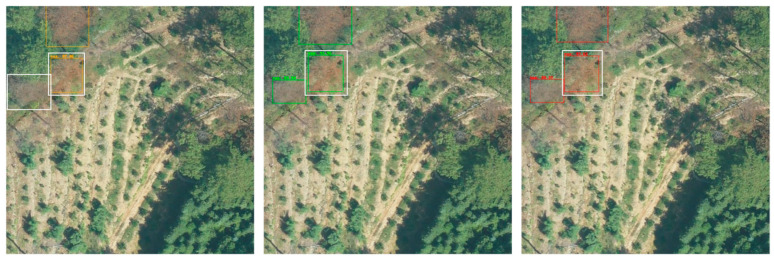

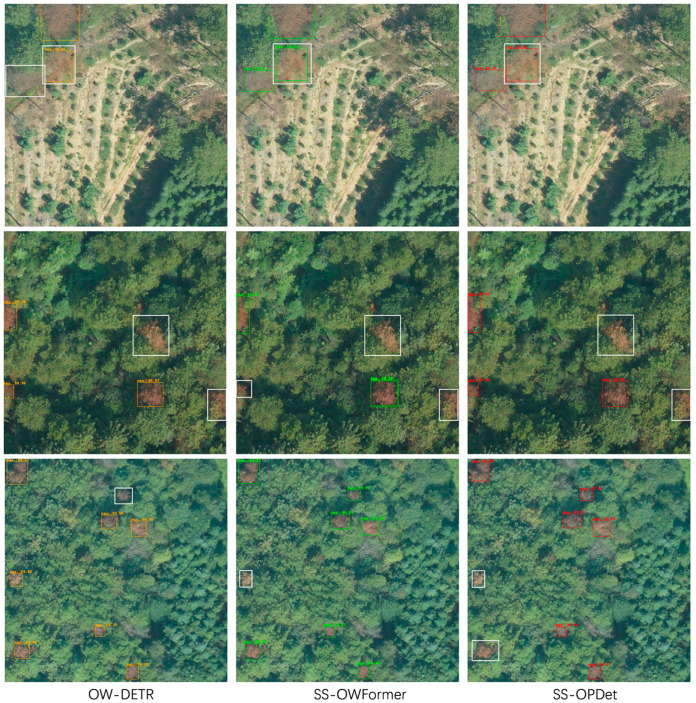
Representative failure cases in dead pine wood detection.

Figure 9 illustrates representative failure cases from three distinct scenes for OW-DETR (left column), SS-OWFormer (middle column), and SS-OPDet (right column). In this figure, orange boxes represent the detection results of OW-DETR, green boxes represent those of SS-OWFormer, and bright red boxes represent the results of SS-OPDet. White bounding boxes are used to highlight regions where all three methods make detection errors. The white bounding boxes indicate regions where all three methods exhibit errors. All models occasionally miss small or partially occluded dead pine wood targets. In addition, unknown objects are sometimes incorrectly detected as dead pine wood due to high visual similarity. While SS-OPDet generally demonstrates superior robustness and precision, these failure cases indicate that the model still encounters challenges under complex conditions. Future work may focus on enhancing sensitivity to small objects, incorporating contextual information, and refining open-set rejection mechanisms to further improve detection reliability.

### 3.2. Ablation Experiments

To systematically evaluate the contribution of each component of SS-OPDet to its overall performance, we conducted a series of ablation experiments on the dead pine wood dataset. The corresponding results are summarized in Table 2. As additional modules are integrated, SS-OPDet exhibits consistent improvements across all evaluation metrics.

Specifically, introducing only the WMFF module raises ${AP}_{K}$ from 82.44% (achieved by SS-OWFormer without WMFF) to 83.41%, and increases $R_{K}$ from 92.81% to 93.16%. Meanwhile, the *AOSE* drops from 305 to 286, indicating that the WMFF module substantially enhances the model’s ability to capture fine-grained features of dead pine wood. This improvement demonstrates that the adaptive weighted fusion of multi-scale features effectively enhances the extraction of detailed target information, particularly in complex forest backgrounds, thereby reducing missed detection and false positives. When only the DCPL strategy is applied, the model’s ${AP}_{K}$ further increases to 83.34%, $R_{K}$ improves to 93.20%, and *AOSE* decreases to 282. These results show that DCPL, by dynamically adjusting the pseudo-label generation process, effectively mitigates the influence of noisy pseudo-labels, enhancing the model’s robustness and stability. In complex scenarios, DCPL facilitates accurate filtering of pseudo-labels, preventing the mislabeling of irrelevant objects as dead pine wood and thereby improving detection outcomes. Finally, when both WMFF and DCPL are used together, SS-OPDet’s ${AP}_{K}$ reaches 84.73%, the $R_{K}$ reaches 94.48%, *AOSE* further drops to 271, and *WI* further drops to 0.0917. These results indicate that the combined effect of WMFF and DCPL not only enhances fine-grained feature extraction but also improves pseudo-label quality and noise suppression. The WMFF module reinforces multi-scale feature fusion to improve precise localization of dead pine wood targets, while the DCPL strategy optimizes the pseudo-label generation process by improving the quality of medium-confidence pseudo-labels. Together, these modules enable the model to efficiently detect known targets and accurately reject unknown ones under open-set conditions, ultimately contributing to superior detection performance.

**Figure 10:**
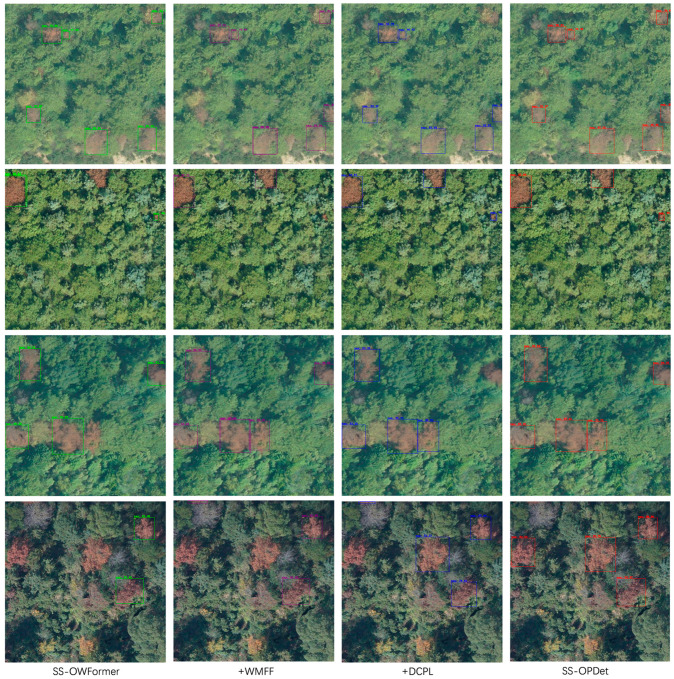
Ablation study of the proposed modules.

Figure 10 presents detection results across four distinct scenes for different configurations of SS-OPDet. In this figure, green boxes represent the detection results of SS-OWFormer, purple boxes represent the results of SS-OPDet with only the WMFF module, blue boxes represent the results with only the DCPL strategy, and bright red boxes represent the full SS-OPDet configuration (with both WMFF and DCPL). Each row denotes a distinct scene, and from left to right, the methods shown are SS-OWFormer, SS-OPDet with only the WMFF module, SS-OPDet with only the DCPL strategy, and the full SS-OPDet (with both WMFF and DCPL). We observe that SS-OWFormer, though able to detect some dead pine wood targets, suffers from significant missed detection and false positives in all scenes, reflecting its limited ability to handle multi-scale targets and suppress unknown interference. When only the WMFF module is applied (second column), the model leverages adaptive multi-scale feature fusion to better capture fine-grained details, resulting in improved detection in some scenes, although certain targets are still missed. With only the DCPL strategy applied (third column), the model filters predictions based on confidence, discarding low-confidence noisy predictions and retaining certain medium-confidence true positives. This configuration partially alleviates the missed detection issue but still struggles with targets at very large or small scales. In contrast, with both WMFF and DCPL integrated (right column, SS-OPDet), the model accurately detects all dead pine wood targets in every scene, with minimal missed detection or false positives. This superior performance can be attributed the efficient multi-scale feature fusion provided by the WMFF module and the dynamic pseudo-label filtering introduced by the DCPL strategy. The synergy between the two modules enables the model to effectively filter out unknown distractors while maintaining focus on known targets, resulting in the best detection performance across all scenes.

### 3.3. Experiments on Different Annotation Ratios

To further explore the potential of the semi-supervised learning strategy in reducing manual annotation effort, we conducted experiments using different annotation ratios. Specifically, we trained SS-OPDet using 75%, 50%, and 25% of the available annotated data, simulating practical scenarios where high annotation cost or data collection challenges limit the availability of labeled samples. It should be noted that in the earlier overall performance comparison experiments, we used 50% of the annotated data. This ratio was selected as it provides sufficient supervision for training while leveraging a substantial amount of unlabeled data, thereby enabling cost-effective detection without significant performance degradation.

As shown in Table 3, when 75% of the data is annotated, SS-OPDet achieves a known-class average precision (${AP}_{K}$) of 87.42%, a recall ($R_{K}$) of 95.63%, and an *AOSE* of 250. This suggests that a high annotation ratio enables the model to achieve optimal detection performance. Reducing the annotation ratio to 50% results in an ${AP}_{K}$ of 84.73%, an $R_{K}$ of 94.48%, and an *AOSE* of 271. Although there is a slight performance drop compared to the 75% scenario, the overall detection capability remains relatively stable. When the annotation ratio is further reduced to 25%, ${AP}_{K}$ decreases to 83.89%, $R_{K}$ to 93.43%, and *AOSE* increases to 278, which shows that under low-annotation conditions, the model’s performance declines somewhat but still remains relatively satisfactory.

Overall, SS-OPDet maintains strong detection performance across varying annotation ratios and effectively utilizes unlabeled data to compensate for limited supervision. This significantly reduces the requirement for extensive manual labeling, offering an efficient and practical solution for large-scale forest monitoring in situations where data annotation is challenging. These findings also validate the use of a 50% annotation ratio as a representative setting, as it achieves a favorable balance between detection performance and annotation cost.

**Figure 11:**
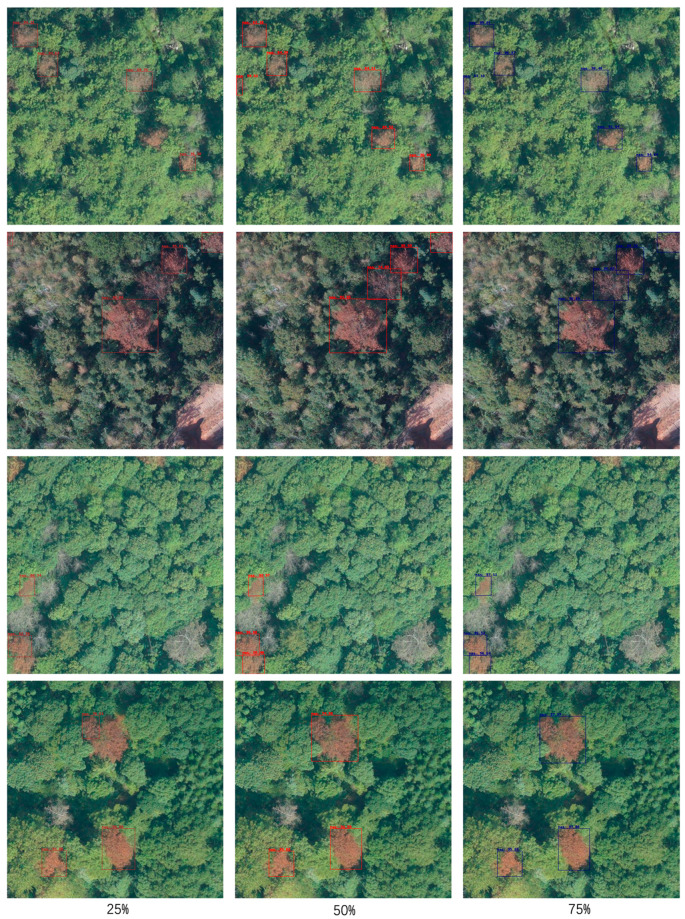
Detection results at different annotation ratios.

Figure 11 provides a visual comparison of detection results across four scenes under the three annotation ratios (75%, 50%, 25%), for a total of 12 images (each row corresponds to a scene and each column to an annotation ratio). In this figure, dark red boxes represent the detection results under the 25% annotation ratio, bright red boxes represent the results under 50%, and dark blue boxes represent the results under 75%. At the 75% annotation ratio (third column), the model accurately detects all dead pine wood targets in each scene, demonstrating strong recognition capability for the known class when sufficient labeled data is available. With 50% annotation (second column), the model still accurately detects all targets, with only a slight decline in overall detection performance. However, when the annotation ratio is reduced to 25% (first column), missed detection increases noticeably, and the overall detection performance is adversely affected. Based on these observations, the 50% annotation ratio was selected as the representative setting for our experiments, as it achieves an optimal balance between detection performance and annotation cost.

### 3.4. Cross-Region Testing Experiments

To assess the generalization ability of SS-OPDet across different geographic regions and imaging conditions, a series of cross-region testing experiments were designed. The entire dataset was divided into five non-overlapping subsets based on geographical location, with each region containing images acquired under diverse conditions. This partitioning ensured both diversity and representativeness of the dataset, providing comprehensive test scenarios to evaluate the model’s adaptability to varying environmental conditions.

In these experiments, a leave-one-region-out cross-validation strategy was adopted, wherein each region was sequentially used as the test set while the remaining regions served as training and validation data. This approach effectively mitigates the influence of intra-region spatial autocorrelation, ensuring that the model is evaluated on entirely unseen regional data. Each training set included diverse environmental characteristics, while the corresponding test set represented real-world conditions in an unseen region, enabling a thorough assessment of SS-OPDet’s robustness and detection performance.

We focused on known-category average precision (${AP}_{K}$) and a known-class recall ($R_{K}$) as the primary evaluation metrics to examine the model’s ability to detect known targets and reject unknown ones. Due to the large variation in the number of unknown objects across regions, *AOSE* was excluded from evaluation, as it could lead to inconsistent comparisons. Table 4 summarizes the detailed results of the cross-region evaluation. Notably, in the “Putian” region, SS-OPDet achieved an ${AP}_{K}$ of 76.13% and an $R_{K}$ of 87.03%, which are lower than the results in the other regions (for instance, in the “Baisha,” “Hongwei,” “Ganzhe,” and “Zhuqi” regions, ${AP}_{K}$ ranged from 84.81% to 87.64% and $R_{K}$ from 95.11% to 97.22%). This performance gap is primarily attributed to the Putian region having a higher density of dead pine wood targets, more challenging acquisition conditions, and a greater repetition of similar scenes, which make it more difficult for the model to capture sufficient distinctive features of the targets.

Furthermore, when averaging the results across all five regions, the overall ${AP}_{K}$ and recall were 84.27% and 94.46%, respectively. These values are highly consistent with the performance on the mixed (pooled) dataset (SS-OPDet achieved 84.73% ${AP}_{K}$ and 94.48% recall, with *AOSE* 271 on the mixed test set). This consistency further demonstrates the applicability of SS-OPDet in real-world forest environments and highlights its ability to maintain high detection accuracy under diverse conditions and environmental variations.

## 4. Discussion

The experimental results above confirm that SS-OPDet significantly outperforms existing methods and effectively addresses the challenges of semi-supervised open-set dead pine wood detection. In the overall performance comparison, SS-OPDet achieved higher precision and recall than both OW-DETR and SS-OWFormer, indicating its superior ability to suppress unknown interference and reduce missed detection. The qualitative and quantitative comparisons show that the new modules we introduced (WMFF and DCPL) play a crucial role in these improvements: the WMFF module enhances the model’s ability to detect targets of varying sizes by fusing multi-scale features, while the DCPL strategy improves training by filtering out low-confidence pseudo-labels. The ablation study further confirms that each module independently contributes significantly to performance—either module alone yields notable improvements in detection accuracy, while their combination produces a synergistic effect that enables SS-OPDet to surpass the baseline models by a substantial margin.

In addition, the experiments with different annotation ratios highlight the robustness and practicality of the semi-supervised approach employed in SS-OPDet. Even with only 25% of the data annotated, the model maintained relatively high precision and recall, with only a slight degradation compared to the 50% and 75% annotation settings. These results demonstrate that SS-OPDet can effectively leverage unlabeled data to compensate for limited labeled samples, a desirable property in real-world scenarios where annotations are expensive or difficult to obtain. This underscores the advantage of the proposed semi-supervised strategy in significantly reducing manual labeling effort without severely compromising detection performance.

The cross-region testing experiments further demonstrate the strong generalization ability of SS-OPDet. The model maintained high detection accuracy across geographically distinct regions with different environmental conditions, and the average performance in cross-region tests closely matched that on the mixed dataset. This consistency indicates that SS-OPDet generalizes well to previously unseen regions, which is critical for practical deployment in large-scale forest monitoring. Although performance in one particularly challenging region (Putian) was somewhat lower due to dense targets and difficult imaging conditions, the method still achieved reasonably good results there, and such findings provide insights into potential areas for future improvement (e.g., handling very dense target scenarios). Overall, these results demonstrate that SS-OPDet effectively meets its design goals: it significantly improves detection of dead pine wood under open-set conditions and limited supervision, reducing false positives and missed detections in complex real-world environments.

## 5. Conclusions

In this study, we proposed SS-OPDet, a semi-supervised open-set dead pine wood detection framework, designed to address the limitations of traditional closed-set detectors in complex forest environments, especially their susceptibility to unknown object interference and missed detection. SS-OPDet effectively leverages a limited amount of annotated data in combination with a large pool of unlabeled samples. It incorporates a Weighted Multi-scale Feature Fusion module to adaptively integrate multi-scale features, thereby enhancing the extraction of fine-grained characteristics of dead pine wood. In parallel, a Dynamic Confidence Pseudo-Label Generation strategy stratifies pseudo-labels by confidence level, effectively filtering out low-confidence noise and reducing both false positives and missed detection. Experimental results demonstrate that SS-OPDet achieves high precision and recall on standard test sets, and also exhibits strong robustness and generalization capability in cross-regional evaluations, adapting well to diverse acquisition conditions across various forest areas. Furthermore, experiments under varying annotation ratios confirm that the semi-supervised strategy offers clear advantages in reducing annotation demands without significantly compromising performance. Overall, this work provides an efficient, cost-effective, and practical solution for the timely detection and management of pine wilt disease, while also opening new research directions for applying semi-supervised open-set detection techniques to remote sensing image analysis. Future work will focus on further optimizing the SS-OPDet architecture and exploring its integration with advanced deep learning techniques. The ultimate goal is to achieve improved detection performance with reduced reliance on annotated data, thereby facilitating the practical application of this approach in large-scale forest monitoring.

## Figures and Tables

**Table 1:** Performance comparison of methods. ↓ indicates that lower values are better, while ↑ indicates that higher values are better.

Method	Labeling Ratios	AOSE(↓)	${AP}_{K}(↑)$	$R_{K}(↑)$	*WI*
OpenDet	100%	386	86.48	93.22	2.6406
Grounding DINO	100%	352	87.35	94.12	2.2875
OW-DETR	50%	433	81.34	93.16	0.2131
SS-OWFormer	50%	305	82.44	92.81	0.0958
SS-OPDet	50%	271	84.73	94.48	0.0917

**Table 2:** SS-OPDet ablation experiment results. The symbol “*” indicates that the corresponding module is not used, while “√” indicates that the corresponding module is used. ↓ indicates that lower values are better, while ↑ indicates that higher values are better.

Method	+WMFF	+DCPL	AOSE(↓)	${AP}_{K}(↑)$	$R_{K}(↑)$	*WI*
SS-OWFormer	*		305	82.44	92.81	0.0958
SS-OPDet	√	*	286	83.41	93.16	0.1181
	*	√	282	83.34	93.20	0.0985
	√	√	271	84.73	94.48	0.0917

**Table 3:** Performance of SS-OPDet at different annotation ratios. ↓ indicates that lower values are better, while ↑ indicates that higher values are better.

Labeling Ratios	AOSE(↓)	${AP}_{K}(↑)$	$R_{K}(↑)$	*WI*
75%	250	87.42	95.63	0.1097
50%	271	84.73	94.48	0.0917
25%	278	83.89	93.43	0.0997

**Table 4:** Cross-region testing results. ↓ indicates that lower values are better, while ↑ indicates that higher values are better.

Test Area	${AP}_{K}(↑)$	$R_{K}(↑)$
Putian	76.13	87.03
Baisha	84.81	95.11
Hongwei	85.54	96.02
Ganzhe	87.25	97.22
Zhuqi	87.64	96.91
Average	84.27	94.46

## Data Availability

The dataset utilized in this study was created by the authors.
